# Bibliographical Notices

**Published:** 1838-05-09

**Authors:** 


### BIBLIOGRAPHICAL NOTICES.

Traité Pratique Des Maladies Vénériennes, ou, *Recherches Critiques et Experimentales sur l'Inoculation appliquée à l'étude de ces Maladies; Suivies d'un Résumé Thérapeutique et d'un Formulaire Spécial.* Par Ph. Ricord, Docteur en Médécine, Chirurgien de l'Hopital des Vénériens de Paris, &c. &c. Paris: 1838, pp. 808, 8vo.

A Practical Treatise on Venereal Diseases, *or, Critical and Experimental Researches upon Inoculation applied to the study of these Diseases,* &c. By Ph. Ricord, M. D., Surgeon to the Venereal Hospital at Paris, &c. &c. Paris: 1838, pp. 808, 8vo.

(Concluded from page 146.)

We next take up the consideration of the author's views on the treatment of venereal affections. Before entering upon them, the subject of the prophylactic treatment of syphilis claims notice. This is very freely treated. The Gordian knot of the moralist M. Ricord cuts at once; he ridicules the idea of viewing syphilis as a scourge from Heaven, for the just punishment of libertinage. We are not disposed to discuss the moral question with the Parisian surgeon, nor to enter into his minutiæ of detail upon the matter of prophylaxis. Police regulations, of a severer character than those even of Paris, he urges as the most probable effectual means of eradicating the Venereal. Such is the view taken by M. Parent Duchatelet, in his work* on the *morale* of the same subject. The introduction, however, of such hygienic regulations into this country would be impracticable.

We shall pass over in rapid review the modes of treatment recommended by the author for the different forms and stages of venereal affections. We shall follow his own division of the subject.

On the subject of *chancres*, his most important inculcation is the necessity of cutting short the disease at its commencement, by getting rid of the seat of infection. Cauterization with the nitrate of silver best effects this object. In the virtue of this article he has great faith, in all stages of the affection, not deeming it incompatible with a high state of inflammation. If the chancre be deeply seated in the tissues, nitrate of silver may not penetrate to a sufficient depth; in these cases the caustic potash is recommended, used with proper precautions. We cannot dwell at length upon the local applications used for ulcerated chancres. They will not be found to differ essentially from the ordinary practice. Much reliance is placed on dressings of charpie, moistened slightly in the French preparation, vin

* De la Prostitution de la Ville de Paris: Paris, 1836.

aromatique. Mercurial ointments are especially eschewed. On the subject of the internal administration of mercurials, the author's views are moderate and consonant with the generally entertained opinions of the day. Admitting that, in the majority of cases, mercury is more hurtful than useful, he grants the existence of circumstances, in which the employment of it may be attended with good results. These, however, he cannot point out with any precision, and, in resorting to the remedy when ordinary means fail, he confesses that he is but yielding to a rational empiricism, out of respect for a therapeutic agent, so long and so often regarded as specific. An exception is made in the case of the indurated phagedenic chancre. Whenever a degree of induration accompanies a chancre, which prevents its cicatrization, or persists, after its superficial cure, and, especially, when it tends to give it a phagedenic form, a mercurial treatment is strongly indicated, particularly as a guarantee against the development of secondary symptoms.

The practical precepts, laid down for the management of *buboes*, are excellent. They offer nothing, however, sufficiently novel, to be presented in detail to our readers. We may mention, that, in the absence of high inflammation, and when the development of a virulent bubo is apprehended, the author's plan is, to cover the tumour with a small blister, and dress the surface with charpie, steeped in a solution of corrosive sublimate, twenty grains to the ounce, or some other caustic, which is kept on for two or three hours, until an eschar is produced. Emollient applications follow, and the ulcer, left by the falling of the eschar, is simply dressed. This must be a painful proceeding, and, we should think, not often admissible.

We pass to the consideration of the treatment of constitutional syphilis. On the subject of prophylaxis, M. Ricord again urges the importance of destroying every sore or solution of continuity that shows itself after copulation, whether its character be deemed suspicious or otherwise. We are confident that a general compliance with this recommendation is the most effectual barrier that can be opposed to the ravages of syphilis. After the establishment of a chancre, the best prophylactic against constitutional infection is the rapid and radical cure of the primitive affection, without remains of induration. If this latter persist, after a mercurial or other treatment, consecutive symptoms may be confidently anticipated. Does the use of mercury favour the development of the syphilitic diathesis? No further, we think, with M. Ricord, than by retarding, if improperly administered, the cure of primary symptoms. To attribute to the effects of mercury the constitutional affections of syphilis, is a much more untenable position, than to urge it as a special preventive or therapeutic agent. Attention to hygienic precepts, by maintaining a proper equilibrium of the functions, and preserving the general health, is a powerful safeguard against the development of secondary symptoms.

The disease having become constitutional, the treatment of the secondary symptoms next occupies our attention. Delay is here obviously inadmissible; under no circumstances, is so grave an affection as syphilis to be allowed to strengthen its hold on the constitution, which it so rapidly undermines. The set of remedies, usually resorted to, comprises antiphlogistics, regimen, baths, sudorifics, tonics, antiscorbutics, and others, which may be comprehended under the general head of alteratives. To all of these M. Ricord attaches due importance. In an antiphlogistic course, properly timed, he has much confidence. The influence of diet is, on all hands, confessedly powerful. The starving system, where there are irritable, inflammatory venereal affections, in robust, vigorous subjects, produces sometimes astonishing effects. Not less happy are the results of proper nourishment, in debilitated individuals, of scrofulous disposition, previously accustomed to scanty and impoverished aliment. The author dwells particularly upon the efficacy of baths in relieving cutaneous affections. In the virtues of sudorifics, and particularly of sarsaparilla, he has little faith. Tonics are never to be neglected; in the Venereal Hospital, daily use is made of bark, the bitters, the martial preparations, particularly the iodide of iron, most valuable in scrofulo-syphilitic affections, and of iodine uncombined. The preparations of gold and silver, lately put forth as almost specifics in syphilis, failed completely in M. Ricord's hands. It is upon *mercurials*, when not counterindicated, that he most relies. Not regarding mercury as a specific, he employs it, in the absence of a specific, as the safest and most powerful remedy we possess. It is to be administered in doses, regulated according to the symptoms and constitution of the patient, and gradually increased till its good effects are produced, or a contrary result points out the propriety of pausing. The increase of doses, M. Ricord has found most efficacious when pushed suddenly from a very small to a very large dose, and after an interval of five or six days, rather than by a gradual daily addition. Salivation is to be avoided, as at best useless, suspending the cure, and adding a serious complication to the existing affection. The preparation preferred by M. Ricord is the protiodide of mercury, commencing with pills of one grain. One other remedy is noticed, opium, which, when specially indicated, or employed as a corrective, during the mercurial course, is highly useful.

The most common secondary symptoms are the eruptions, which appear on the skin and certain mucous membranes. These may vary in character and duration, may terminate simply, or, running into suppuration, become pustules and afterwards ulcers, or, finally, end in the formation of indurated or ulcerated tubercles. For a rational diagnosis of such affections, the author thinks we can look only to the previous existence of a chancre, or to a certainty of hereditary taint. Even the copper colour of the blotches, so generally laid down as characteristic, usually shows itself late, is well marked only after they have been deeply seated in the skin, and is not detected in the mucous membranes. In the treatment of these cutaneous affections, commencing with antiphlogistics, if there be febrile excitement, reliance is mainly to be placed on a mercurial course. Mucilaginous and vapour baths, fumigations with cinnabar, and, in the scurfy and pustular forms, frictions with the ointment of the protiodide of mercury, are highly advantageous. M. Ricord considers his local treatment for the mucous tubercle, (venereal wart,) as specific, so certain and rapid are its curative powers. His plan is to wash the parts twice or thrice, with pure chloride of soda, if not ulcerated, and, if in this condition, with a solution, just strong enough to be felt. Calomel is afterwards sprinkled over them, and, under these applications, enormous masses of the eruptions disappear, in a very few days. Syphilitic ulcers are to be treated much as chancres; when in the throat and other internal situations, M. Ricord employs the ordinary gargles and dressings.

Syphilitic iretis is managed by the usual measures of depletion and counter-irritation, with a mercurial course; sarcocele is treated by mercurials, locally and internally.

Perhaps no class of syphilitic affections is more interesting, than those which the author classifies as *tertiary*. As their diagnosis is obscure, so has their dependence upon primary syphilis been called in question. There are not wanting those, who attempt to trace them, in their most serious and intractable forms, to the remedies, particularly mercurials, used in combating the original affection. This opinion, however, is, we think, not warranted by argument or facts. The ordinary effects of mercury do not certainly show themselves under the hideous aspect, to be met with in the venereal wards of our hospitals; nor can they, who deny the specific nature of syphilis, and treat it as a simple irritation, be allowed to account for these effects, by a supposed complication of the influences of the remedy and the disease. That the worst features of constitutional syphilis have shown themselves in cases, not treated by mercury, is a well established fact; while the number of individuals is not small, who, although exposed to the full action of this medicine, have recovered without any such after symptoms. The diagnosis of the tertiary affections is established, by the frequency of their occurrence after primary syphilis, by the absence of other causes, and by their development after characteristic secondary symptoms, which are almost invariably the connecting link between the primary and tertiary appearances. On the therapeutics of special tertiary affections, we cannot go into particulars. It is sufficient to remark, that M. Ricord has not here the same confidence in the powers of mercury, as in the secondary form. His plan of treatment varies with the symptoms, and seems judicious.

The remaining chapters of the work, on the various forms of blennorrhagia, and some kindred affections, we should be glad to be able to notice fully; but this review has been already extended beyond our ordinary limits. We have wished, however, to bring before our readers a detailed account of the novel views of M. Ricord, on a topic, which, much as it has been discussed, has lost none of its interest. An experimental work, announcing important conclusions, the Traité Pratique has strong claims to notice. It throws light upon many obscure points, and establishes, upon the basis of observation, the moderate doctrines, on the vexed subject of syphilis, upon which medical opinions have now generally converged. We have preferred presenting a simply analytical rather than a critical review of such a work.

---

*Transactions of the Medical Society of the State of New York.* Vol. IV. Part I. *Albany*, 1838, p. 80.

This is an interesting pamphlet, offering, however, but little variety of matter. We have read with pleasure the address on homœopathy by Dr. McNaughton, the president of the society, the tone of which is characterized by good sense and moderation. A commendatory notice of Dr. Hull's "Utero Abdominal Supporter," by Dr. John F. Gray, of New York, and Statistics of the Blind, by S. Hazard, Esquire, of Philadelphia, are the remaining papers of the transactions. We could wish to see a portion of the vitality of the New York Medical Society imparted to some of its lethargic sisters.

